# Anti-Metastatic Properties of a Marine Bacterial Exopolysaccharide-Based Derivative Designed to Mimic Glycosaminoglycans

**DOI:** 10.3390/molecules21030309

**Published:** 2016-03-04

**Authors:** Dominique Heymann, Carmen Ruiz-Velasco, Julie Chesneau, Jacqueline Ratiskol, Corinne Sinquin, Sylvia Colliec-Jouault

**Affiliations:** 1 INSERM, UMR957, Institut National de la Santé et de la Recherche Médicale, Unité Mixte de Recherche 957, Laboratoire de Physiopathologie de la Résorption Osseuse et Thérapie des Tumeurs Osseuses Primitives, Equipe Ligue Contre le Cancer 2012, Nantes 44035, France; dominique.heymann@sheffield.ac.uk (D.H.); ruizvela@usc.edu (C.R.-V.); julie.chesneau@laposte.net (J.C.); 2 IFREMER, Institut Français de Recherche pour l’Exploitation de la Mer, Laboratoire EM^3^B Ecosystèmes Microbiens et Molécules Marines pour les Biotechnologies, BP21105, Nantes 44311, France; Jacqueline.Ratiskol@ifremer.fr (J.R.); Corinne.Sinquin@ifremer.fr (C.S.)

**Keywords:** exopolysaccharides, glycosaminoglycan, heparin-like, derivatives, sulfation, bone metabolism, bone remodeling, lung mestatases, osteosarcoma

## Abstract

Osteosarcoma is the most frequent malignant primary bone tumor characterized by a high potency to form lung metastases. In this study, the effect of three oversulfated low molecular weight marine bacterial exopolysaccharides (OS-EPS) with different molecular weights (4, 8 and 15 kDa) were first evaluated *in vitro* on human and murine osteosarcoma cell lines. Different biological activities were studied: cell proliferation, cell adhesion and migration, matrix metalloproteinase expression. This *in vitro* study showed that only the OS-EPS 15 kDa derivative could inhibit the invasiveness of osteosarcoma cells with an inhibition rate close to 90%. Moreover, this derivative was potent to inhibit both migration and invasiveness of osteosarcoma cell lines; had no significant effect on their cell cycle; and increased slightly the expression of *MMP-9*, and more highly the expression of its physiological specific tissue *inhibitor TIMP-1*. Then, the *in vivo* experiments showed that the OS-EPS 15 kDa derivative had no effect on the primary osteosarcoma tumor induced by osteosarcoma cell lines but was very efficient to inhibit the establishment of lung metastases *in vivo*. These results can help to better understand the mechanisms of GAGs and GAG-like derivatives in the biology of the tumor cells and their interactions with the bone environment to develop new therapeutic strategies.

## 1. Introduction

Osteosarcoma is the most frequent malignant primary bone tumor that occurs mainly in the young, with an incidence peak observed at 18 years. Despite recent improvements in chemotherapy and surgery, the problem of non-response to chemotherapy remains and this poor prognosis warrants new therapeutic strategies to improve the overall rate of survival. The osteosarcoma is characterized by a high potency to form lung metastases that is the main cause of death [1,2]. Recent studies have described the molecular mechanisms of metastasis occurrence that can help to identify new therapeutic strategies [3]. Carbohydrates and especially heparin or heparan sulfate are now considered as good candidates to treat cancers, in particular cancer metastasis, but nevertheless their therapeutic use is limited because they present both anticoagulant activity and consequently they can induce adverse bleeding complications. Further disadvantages of heparin and heparan sulfate are their animal origin with a high risk of unknown cross-species contamination [4,5]. Consequently, the exploration of the therapeutic potential of heparin mimetics is now booming. Sulfated oligosaccharides are currently studied such as a sulfated form of phosphomannopentaose and phospohomannotetraose named PI-88 [6], sulfated form of maltohexose and sulfated maltotriose [7]. Recently, two polysaccharides extracted from *Prunella vulgaris* L. were described for their anti-lung adenocarcinoma activity [8].

In recent years, there has been a growing interest in the isolation and identification of new microbial polysaccharides that might have new uses in many industries. They compete with polysaccharides from other sources such as seaweeds, crustaceans, animals or plants. Interest in mass culture of microorganisms from the marine environment has increased considerably, representing an innovative approach to the biotechnological use of under-exploited resources. When sulfated, polysaccharides from different sources can share some biological properties with glycosaminoglycans and especially heparan sulfate or heparin without presenting the same bleeding risk and with a low risk to be contaminated by a non-conventional transmissible agent such as prions or emerging viruses due to a large “species-barrier” [9].

Marine bacteria associated with deep-sea hydrothermal conditions have demonstrated their ability to produce, in an aerobic carbohydrate-based medium, unusual extracellular polymers. They present original structural features that can be modified to design bioactive compounds and improve their specificity [10,11]. In particular, with the aim of promoting biological activities, chemical modifications (depolymerization and substitution reactions) of one exopolysaccharide (GY785 EPS) produced by a deep-sea hydrothermal bacterium named *Alteromonas infernus* have been undertaken. A low molecular weight (LMW) oversulfated derivative (OS-EPS) has been isolated after chemical modifications of this native GY785 EPS. This derivative is less efficient (10 fold) than heparin in clotting assays. In activated partial thromboplastin time, the same anticoagulant effect was obtained at 10 µg/mL and 1.5 µg/mL of OS-EPS derivative and heparin, respectively [12]. The structure of the native GY785 EPS has been described [13].

The growth and differentiation of bone cells is controlled by various factors that can be modulated by heparan sulfates. The heparan sulfate effect is not only charge dependent but also chain length dependent. The results of clinical trials indicated that LMW GAGs had less effect on bone formation than unfractionated GAG [5]. The effects of the derivative named OS-EPS on bone biology have been previously studied. The effect of this highly sulfated LMW derivative (40% sulfate groups and 24 kDa) has been compared with that of a non-oversulfated LMW GY785 EPS (10% sulfate groups and 13 kDa). The observed data have shown different levels of bone resorption regulation by GAGs or OS-EPS, most of them leading to pro-resorptive effects [14].

The chain length impact of OS-EPS derivatives on several kinds of biological activities was investigated here to determine which size could be the most effective on bone tumor growth model. First, in some *in vitro* experiments, we compared the activity of three OS-EPS derivatives with various molecular weights (4, 8 and 15 kDa) on osteosarcoma cell lines (mouse POS-1 and human HOS cells), and using heparin as a reference. Proliferation, migration, cell cycle analysis and expression in osteosarcoma cell lines of matrix metalloproteinases such as gelatinases MMP-2 and MMP-9 and their inhibitors TIMP-1 and TIMP-2 were studied. Then, the OS-EPS derivative (OS-EPS 15 kDa) showing the most interesting properties *in vitro* was evaluated *in vivo* on both primary malignant bone tumor growth (paratibial model) and establishment of lung metastases in osteosarcoma mouse model, and again heparin was used as a reference.

## 2. Results and Discussion

### 2.1. Characterization of OS-EPS Derivatives

The molecular weight, polydispersity and chemical composition of each derivative are presented in Table 1. LMW OS-EPS derivatives were homogeneous with a low polydispersity. After the chemical oversulfation, no important change in their respective initial osidic composition was observed. The OS-EPS derivatives had a sulfur content above 10%, corresponding to above 30% of sulfate groups close to that found in heparin.

### 2.2. In Vitro Effect of OS-EPS on Osteosarcoma Cell Lines

#### 2.2.1. Cell Proliferation and Cell Viability

**Figure 1:**
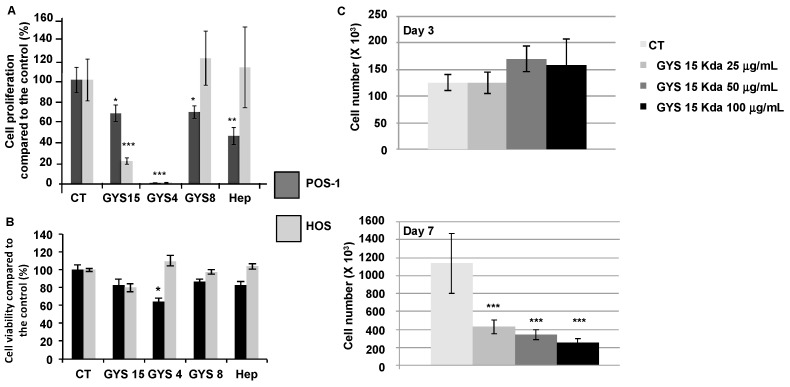
Comparison of the effects of OS-EPS derivatives with various molecular weights on two osteosarcoma cell lines, the mouse POS-1 and human HOS: (**A**) proliferation of both cell lines after seven days of treatment; (**B**) cell viability assessment of HOS and POS-1 cells after three days of treatment with or without OS-EPS derivative treatment; and (**C**) kinetic of biological activity of increasing doses of GYS15 on HOS cell proliferation. Proliferation assays were performed by cell counting with Trypan Blue to compare the cell proliferation rate between groups. OS-EPS 15 kDa (GYS15), OS-EPS 8 kDa (GYS8), OS-EPS 4 kDa (GYS4), heparin (Hep) and control (CT). * *p* < 0.05; ** *p* < 0.01; *** *p* < 0.001.

Compared to the controls, only OS-EPS 4 kDa and 15 kDa derivatives significantly inhibited both mouse POS-1 and human HOS cell proliferation. The most potent OS-EPS derivative to inhibit the proliferation of osteosarcoma cell lines was the OS-EPS 4 kDa (Figure 1A). After three days of treatment with GYS15 no significant effect was shown on HOS cell proliferation or cell viability in contrast to GYS4 (Figure 1B). After seven days of treatment, 25 µg/mL of GYS15 markedly decreased the HOS cell proliferation (Figure 1C). These data are in agreement with those obtained on osteoblastic cells [14].

#### 2.2.2. Cell Migration Assay

**Figure 2:**
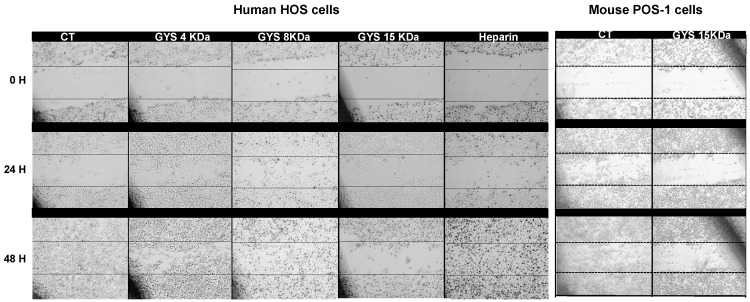
Migration of the human HOS cells and mouse POS-1 cells in the presence of various OS-EPS derivatives (300 µg/mL): OS-EPS 15 kDa (GYS15), OS-EPS 8 kDa (GYS8), OS-EPS 4 kDa (GYS4), heparin and control (CT). This experiment was repeated three times and a representative experiment is shown.

We investigated the effects of OS-EPS derivatives on the osteosarcoma cell migration using an *in vitro* wound-healing assay. All compounds assessed (OS-EPS4, OS-EPS8, OS-EPS15 and heparin) inhibited the migration of murine POS-1 osteosarcoma cells (data not shown). In contrast to this cell line, only OS-EPS 4 and 15 kDa were potent to inhibit the migration of human HOS osteosarcoma cells (Figure 2). Indeed, OS-EPS 15 kDa derivative strongly slowed down the migration of HOS cells compared to the other compounds.

It is admitted that heparin, which is conventionally administered as an anticoagulant, has a variety of additional biological activities especially on cancer cells [15,16]. *In vitro* and *in vivo* experimental evidence demonstrated that heparin is an efficient inhibitor of cell migration, adhesion and metastasis [15]. Common molecular pathways with platelet-tumor cell thrombi formation such as the inhibition of heparanase or P-/L-selectin may be involved in this activity [17]. In contrast to heparin, OS-EPS derivatives assessed in the present work inhibited the cell migration of osteosarcoma, suggesting a mechanism of action independent of heparanase and selectin. Mechanisms associated with inhibition of integrin activity can be hypothesized [18].

#### 2.2.3. Cell Invasion Assay

**Figure 3:**
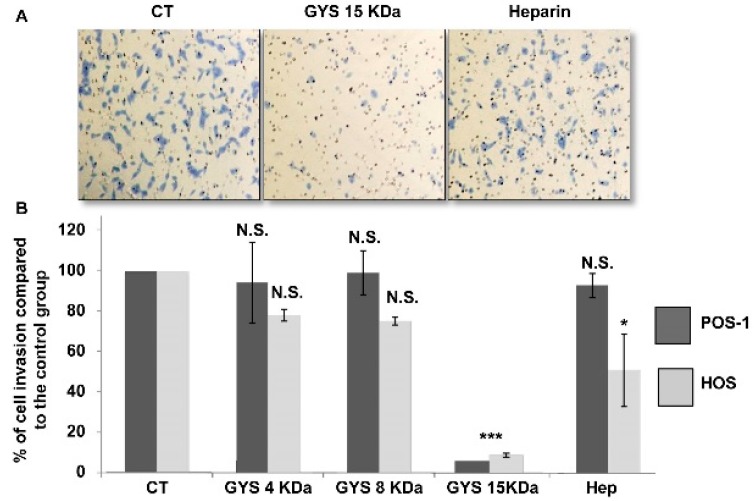
Invasion of the osteosarcoma cell lines, the mouse POS-1 and human HOS, in the presence of OS-EPS derivatives with various molecular weights: 25 µg/mL OS-EPS 15 kDa (GYS15), 50 µg/mL OS-EPS 8 kDa (GYS8), 50 µg/mL OS-EPS 4 kDa (GYS4), 50 µg/mL heparin (Hep) and control (CT). (**A**) Microscopic photographs of invasive HOS cells treated or not with of GYS15 or heparin; (**B**) Cells migrating through the Boyden’s chambers were counted in five microscopic fields using Image J software. N.S.: not statistically significant; * *p* < 0.05; *** *p* < 0.01.

Contrary to the other compounds, only the OS-EPS 15 kDa derivative inhibited the invasiveness of osteosarcoma cells with a inhibition rate close to 90% after 24 h (Figure 3A). This effect was observed for both mouse POS-1 and human HOS at a concentration of 25 µg/mL (Figure 3B). OS-EPS 4 kDa and 8 kDa had no effect up to 200 µg/mL (highest concentration tested).

#### 2.2.4. Cell Cycle Analysis

**Figure 4:**
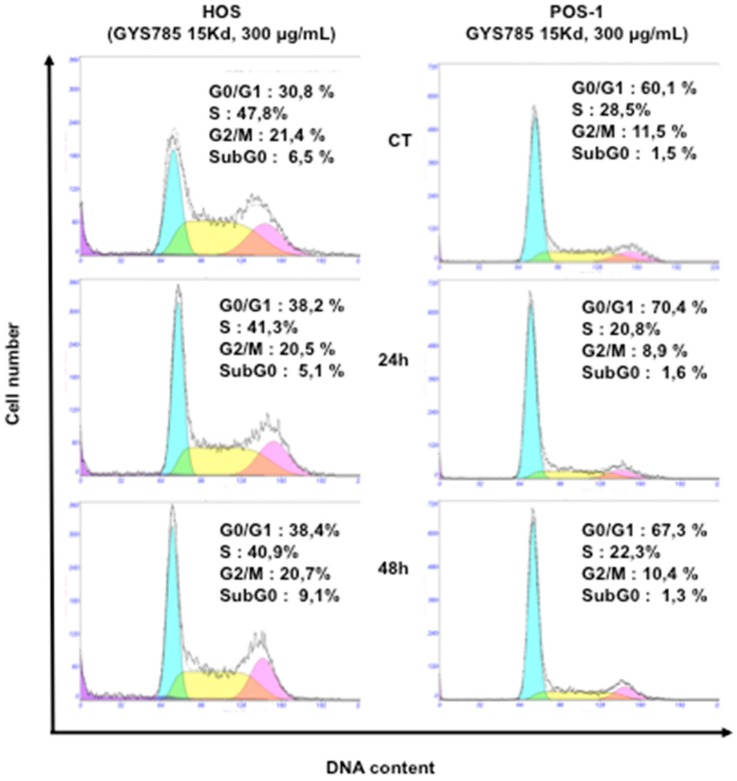
Effect of the OS-EPS 15 kDa derivative (300 µg/mL) on the cell cycle of osteosarcoma cell lines: cell cycle distribution of human HOS and mouse POS-1 cells were studied by flow cytometry after 24 h and 48 h treatment with OS-EPS derivatives. Experiments were repeated three times and a representative experiment is shown.

At the concentration of 300 µg/mL, the OS-EPS 15 kDa derivative had no significant effect on the cell cycle of both mouse POS-1 and human HOS (Figure 4). In addition, heparin also had no effect on the cell cycle at the same concentration (data not shown). Similar data were obtained after 72 h of treatment (data not shown).

#### 2.2.5. Expression in Human HOS Osteosarcoma Cell Line of Matrix Metalloproteinases (MMPs) and Their Inhibitors (TIMPs)

**Figure 5:**
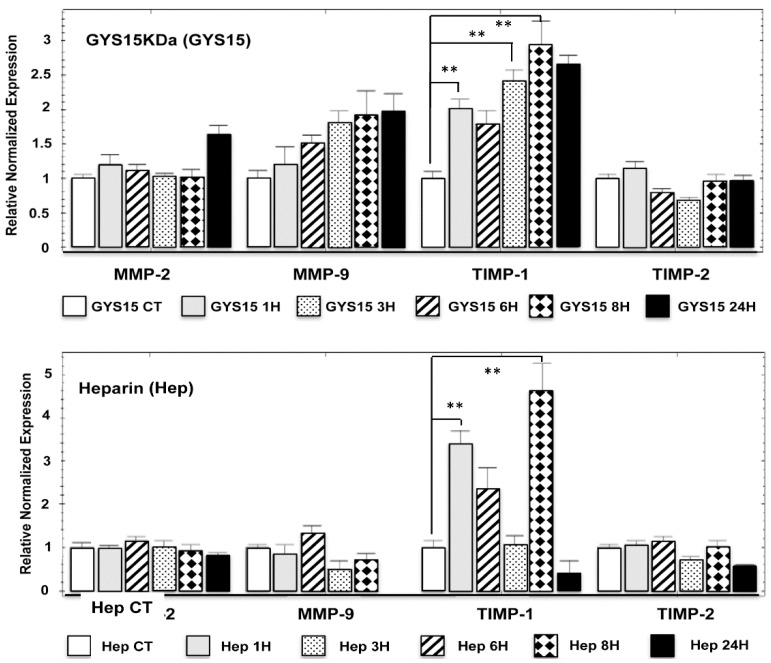
Effect of OS-EPS 15 kDa derivative on the expression of *MMP-2* and *MMP-9* and their inhibitors (*TIMP-1* and *TIMP-2*, respectively) compared to heparin on human HOS cell line. The osteosarcoma cells were treated 1, 3, 6, 8 and 24 h with OS-EPS 15 kDa derivative or heparin at the concentration of 50 µg/mL or not (control). The cell expression of MMPs and their inhibitors in osteosarcoma cells was determined by RT-qPCR. ** *p* < 0.01.

Because OS-EPS 15 kDa derivatives modulated human HOS osteosarcoma cell migration and invasion, we analyzed the expression of the main key regulators of these processes, especially MMPs and their TIMP inhibitors. As expected, human HOS osteosarcoma cells expressed *MMP-2* and *MMP-9* as well as their inhibitors *TIMP-1* and *TIMP-2* (Figure 5). *MMP-2* and its inhibitor (*TIMP-2*) were not modulated by the treatment with OS-EPS derivatives or by heparin. On the contrary, *MMP-9* expression was increased by OS-EPS 15 kDa in a time-dependent manner. Indeed, 50 µg/mL of GYS 15 kDa induced a two-fold increase of MMP-9 mRNA expression compared to the untreated cells (Figure 5). Simultaneously, GYS 15 kDa upmodulated the expression of *TIMP-1*, a natural inhibitor of *MMP-9* activity. Heparin modulated only the expression of *TIMP-1* (Figure 5).

Whether MMP-9 was linked to the migration, proliferation and invasiveness of osteosarcoma cells [19,20], the use of MMP-9 as biomarker of survival in patients with osteosarcoma remains controversial [21,22]. Nevertheless, part of the anti-invasion and anti-migration of OS-PES 15 kDa derivative can be explained by the alteration of protease regulation [23]. This dysregulation of protease expression may be also favor inhibition of tumor apoptosis [24,25].

### 2.3. In Vivo Studies

#### 2.3.1. Primary Malignant Bone Tumor Growth

**Figure 6:**
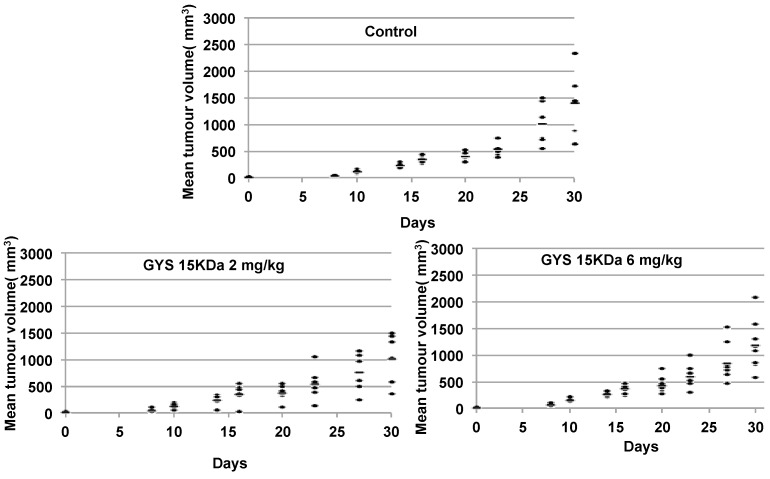
Effect of OS-EPS 15 kDa derivative on the *in vivo* HOS osteosarcoma tumor growth: 2 × 10^6^ HOS cells were inoculated in paratibial area. When tumor volume reached 100 mm^3^, OS-EPS 15 kDa (2 or 6 mg/kg daily) was injected subcutaneously each day and the tumor growth was measured from Day 5 to Day 30.

A curative treatment was performed on mouse model (paratibial model) of osteosarcoma induced by inoculation of mouse POS-1 or human HOS cell line. The treatment started when the tumor volume reached 100 mm^3^ (Day 0) and the mice were divided in three groups: 1—treated with PBS (control); 2—treated with OS-EPS 15 kDa derivative; and 3—treated with heparin. The polysaccharides were injected subcutaneously (50 µL) each day at 2 or 6 mg/kg and the tumor growth was measured from Day 5 to Day 9. Mouse body weights, measured twice a week, were equivalent in the three groups studied, demonstrating that polysaccharides had no impact on the body weight (data not shown). Both polysaccharides were not able to inhibit primary tumor growth in preclinical osteosarcoma mouse models. These results demonstrated that both OS-EPS 15 kDa derivative and heparin had no effect on the primary osteosarcoma tumor induced by either mouse POS-1 cell line (Figure 6) or by human HOS cell line (Figure 6). OS-EPS derivative had no pro-apoptotic effect on osteosarcoma cells as analyzed by terminal deoxynucleotidyl transferase dUTP nick end labeling staining (TUNEL) (Supplementary Figure S1). This result is an agreement with the increase of TIMP-1 expression after OS-EPS stimulation, which can protect cancer cells from death [24,25].

#### 2.3.2. Model of Lung Metastases from Mouse Osteosarcoma

**Figure 7:**
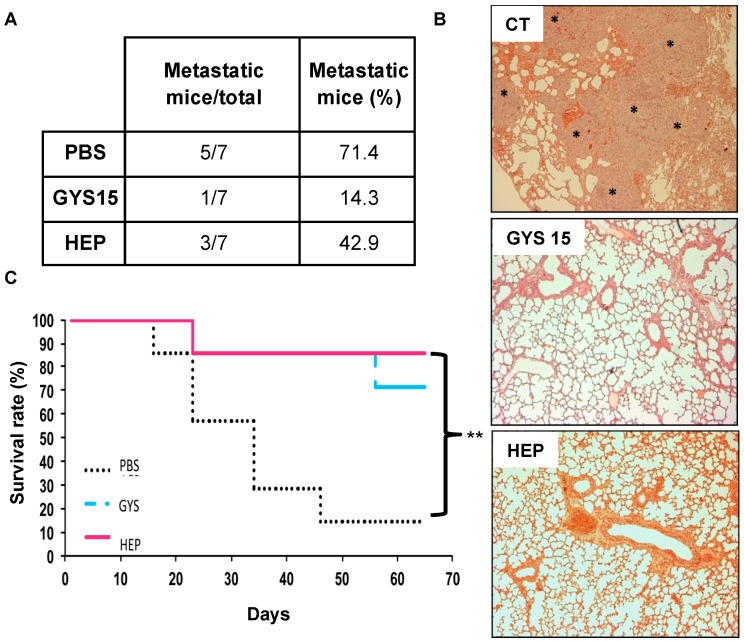
Effect of OS-EPS 15 kDa derivative on the lung metastatic incidence: (**A**) metastatic incidence in treated animals (OS-EPS derivative or heparin; s.c. 6 mg/kg daily) *vs.* control (PBS); (**B**) histological analyses of the lung tissue of treated animals or not (* metastatic foci), original magnification: 400×; and (**C**) survival rate (%) of treated animals (OS-EPS derivative or heparin) compared to the control group (PBS), Kaplan–Meier survival curves. *n* = 7 mice/group; ** *p* < 0.01.

A preventive treatment was establish to study the effect of OS-EPS 15 kDa derivative on the metastatic ability of osteosarcoma by the technique of retro-orbital injection of the venous sinus in mice [23]. In this experiment, mice received POS-1 cells, metastases arising within a few weeks after POS-1 cell injection. OS-EPS 15 kDa derivative treated mice had significantly less metastases (around 40% of decrease) than the untreated ones or heparin treated ones (*p* < 0.001) (Figure 7A). The histological analyses of lung tissue showed that OS-EPS 15 kDa derivative treated mice did not exhibit the presence of metastatic foci similarly to heparin in contrast to the control group treated with a vehicle (PBS) (Figure 7C). This lower incidence of detectable lung metastases was accompanied by an improvement of animal survival rate: 70% of treated animal survived 65 days after the POS-1 cell line injection, whereas only 14% of the control group survived (Figure 7C). As expected, heparin decreased the incidence of lung metastatic incidence [15,16]. No adverse effect of OS-EPS derivatives was observed in mice. In addition to heparin [15,16], various polysaccharides were already envisaged in the treatment of solid cancers [26]. OS-EPS derivatives such as GYS 15 kDa, belongs to these compound family. Recently, a polysaccharide isolated from *Prunella vulgaris* L. (PV), a plant often utilized in traditional Chinese medicine, showed antitumor activity in a pre-clinical model of lung adenocarcinoma [8]. GYS 15 kDa exhibits anti-metastatic activity and its low efficiency in clotting assays [13,14], is clearly an added therapeutic value. OS-EPS effect on metastatic process may also be strengthened by the increase of TIMP-1 expression, which was shown to enhance tumor kinetic and angiogenesis and was also responsible for the creation of premetastatic niche by immune cell recruitment. Numerous hypotheses can be proposed to explain the effects of heparin in absence of effect on cell migration and invasion [27,28]. Among these hypotheses, TRAIL (TNF Related Apoptosis Inducing Ligand) is spontaneously produced by immune cells in response to tumor invasion and to kill cancer cells. TRAIL activities can be blocked by osteoprotegerin (OPG) which is produced by tumor cells [29]. Therein, OPG can act as an anti-apoptotic and a pro-proliferative factor for cancer cells by blocking TRAIL activity in the control group [30]. However, OPG possesses a heparin-binding domain which is known to regulate OPG and glycoaminoglycans and proteoglycans are able to inhibit OPG activity reinforcing TRAIL activity similarly to the heparin group [31]. Overall, activity levels observed of OP-EPS and heparin can be explained by their differential affinities to OPG and other proteins with heparin-binding domain.

## 3. Experimental Section

### 3.1. General

The bacterial GY785 EPS was produced, purified and characterized as previously described [32,33]. The preparation, purification and characterization of oversulfated (OS) low molecular weight (LMW) EPS derivatives (OS-EPS 15 kDa (GYS15), OS-EPS 8 kDa (GYS8) and OS-EPS 4 kDa (GYS4)) have been reported previously [14,33,34]. Briefly, native high molecular weight (HMW) GY785 EPS was depolymerized first using a free-radical depolymerization process to obtain LMW derivatives at different molecular weights. LMW GY785 EPS derivatives were therefore sulfated in dimethylformamide (DMF) using pyridine sulfate as sulfating agent leading to OS EPS derivatives. Molecular weight (M_W_) before and after sulfation was determined by HPSEC-MALS and sulfur content (wt % S) by both elemental analysis and HPAEC chromatography. ATR-FTIR and NMR spectroscopy were used to assess the efficiency of sulfation reaction. Heparin sodium salt from porcine intestinal mucosa H4784 was purchased from Sigma.

### 3.2. Proliferation Assay

POS-1 cell line was cultured in RPMI (Roswell Park Memorial Institute, Biowhittaker, Castleford, UK) medium with 10% fetal bovine serum (FBS, Hyclone, France). KHOS/NP (HOS, (ATCC, Manassas, VA, USA)) was cultured in DMEM (Dulbecco’s Modified Eagle Medium, Biowhittaker) with 5% FBS. The cells, initially seeded at the concentration of 50 × 10^3^ cellules/cm^2^, were incubated at 37 °C with humidity saturated controlled atmosphere and 5% CO_2_. At confluence, cells were detached with trypsine-EDTA (Biowhittaker, Trypsine: 0.5 g/L; EDTA (Ethylene Diamine Tetraacetic Acid): 0.2 g/L). Trypsine was neutralized by adding FBS containing medium and cells were collected after centrifugation at 400 *g*. POS-1 and HOS cell lines were seeded in triplicate at two thousand cells per well in a 24-well plate with 500 µL of medium and treated in the presence of OS-EPS 4, 8 or 15 kDa derivative (25, 50 ou 100 μg/mL) or not (control). Proliferation assays were performed by manual counting of alive cells using a Malassez cell with Trypan Blue to compare the cell proliferation rate between groups.

### 3.3. Migration Assay

Cells seeded (4 × 10^5^) in 6-well plates in duplicate were treated with mitomycin C (4 μg/mL during 1 h, Sigma Aldrich, Saint Quentin Fallavier, France) to block cell proliferation and the migration of cells was evaluated in the presence or not of OS-EPS 4, 8 or 15 kDa derivative or heparin (25 or 50 µg/mL). At confluence, cells were carefully scratched with the tip to create a gap. Cells Images of the gap width were acquired using an Olympus DP12-2 camera (Olympus Corporation, Tokyo, Japan) after 0, 24, 48 and 72 h of incubation.

### 3.4. Invasion Assay

Invasion of cultured cells (POS-1 and HOS) was analyzed using Boyden’s chambers (8 μm pores, Becton Dickinson Labware, Le Pont-de-Claix, France) covered by polyethylene terephtalate membrane with Matrigel^®^ coating (2 μg/100 μL/well in cold PBS) in 24-wells plate (Multiwell™ 24, FALCON^®^). The OS-EPS 4, 8 or 15 kDa derivative (25, 50,100 or 200 µg/mL) were added on the Matrigel 30 min before cell seeding. Cells previously treated with mitomycin C (4 μg/mL during 1 h, Sigma) were seeded in the upper compartment of 500 μL cups in 1% FBS medium (2 × 10^4^ POS-1 cells or 3 × 10^4^ HOS) and left 24 h for incubation at 37 °C in 5% CO_2_ humidified atmosphere. The bottom wells in the system were filled with 10% FBS medium (700 µL) as a chemoattractant. At the end of the 24 h-period, non-invasive cells were removed with cotton swabs and invading cells present on the inferior surface of the membrane were fixed by 3% PFA (ParaFormAldehyde) and stained by methylene blue. After drying, the invasive cells were counted in 5 microscopic fields using Image J software (version 1.49, NIH, Bethesda, MD, USA). All experiments were done 3 times in duplicates and invasion is expressed by mean number of cells/field.

### 3.5. Cell Cycle Analysis

Cells were incubated for 24, 48 or 72 h in medium containing or not containing OS-EPS derivatives. Cells were incubated during 24, 48 or 72 h in medium containing or not containing OS-EPS derivatives. After the incubation period, trypsinized cells were incubated in phosphate-buffered saline containing 0.12% Triton X-100, 0.12 mmol/L ethylenediamine tetraacetic acid, and 100 μg/mL DNase-free RNase A (Sigma). Then, 50 μg/mL propidium iodide were added, and the cells were incubated for 20 min at 4 °C in the dark. Cell cycle distribution was studied by flow cytometry (Cytomics FC500; Beckman Coulter, Roissy, France) based on 2N and 4N DNA content and analyzed using DNA Cell Cycle Analysis Software (version 306, Phoenix Flow Systems, San Diego, CA, USA) [18].

### 3.6. Matrix Metalloproteinase Expression

Cells were seeded (5 × 10^5^) in petri dishes (diameter of 60 mm) in 3 m of medium with FBS. At confluence, cells were treated 1, 3, 6, 8 and 24 h with OS-EPS 15 KDa derivative or heparin at 50 µg/mL or with PBS (control). Matrix Metalloproteinase (MMP) and Tissue Inhibitors of Metalloproteinase (TIMP) expression was determined by quantitative-polymerase chain reaction (qPCR). RNA was extracted using NucleoSpin RNAII (Macherey Nagel, Duren, Germany) and used for first strand cDNA synthesis using ThermoScript real-time polymerase chain reaction (RT-PCR) System (Invitrogen, Carlsbad, CA, USA). Quantitative-PCR (qPCR) was performed with a Chromo4 instrument (Biorad, Richmond, CA, USA) using SYBR Green Supermix reagents (Biorad).

### 3.7. Animal Ethics

All procedures involving animals were conducted in accordance with the Directive 2010/63/EU of the European Parliament and the Council of the 22/09/2010 on the protection of animals used for scientific purposes. The protocols presented in this study were approved by the French ethics committee (CEEA PdL. 06) with the protocol number 2010.34 and under the supervision of the authorized investigators. Four-week-old male NMRI-Nude mice (*n* = 6) and four-week-old male C3H/HeN mice (*n* = 7) from Elevages Janvier (Le Genest Saint Isle, France) were maintained under pathogen-free conditions at the Experimental Therapy Unit (Faculty of Medicine, Nantes, France) in accordance with the institutional guidelines of the French Ethics Committee (CEEA Pays de la Loire—06).

### 3.8. Osteosarcoma Mouse Model

Four-week-old male NMRI-Nude mice (*n* = 6 per group) were anaesthetised by inhalation of an isoflurane/airmixture (1.5%, 1 L/min) before receiving an intramuscular injection of 2 × 10^6^ HOS cells in the paratibial area (in 50 µL pf PBS buffer) [35]. Similarly, 1.5 × 10^6^ POS-1 cells (*n* = 6 per group) were inoculated in four-week-old female C57BL/6 mice [36]. Tumors appeared at the injection site 8 days later. Tumor volume (V) was calculated from the measurement of two perpendicular diameters using a caliper, according to the following formula: V = 0.5 × L × (S)^2^, in which L and S are, respectively, the largest and smallest perpendicular tumor diameters. A curative protocol was performed, when the tumor volume reached 100 mm^3^ mice were treated with PBS (control) or OS-EPS derivatives. We randomized mice into different groups by tumor volume. The polysaccharides diluted in PBS buffer (50 µL) were injected subcutaneously each day at 2 or 6 mg/kg and the tumor growth was measured from Day 5 to Day 35. This mouse model (paratibial model) was chosen rather than the model using the injection of tumor cells directly into the tibia (intratibial model) because bone lesions were very similar at the end of the experiment in the two models and because the results are more reproducible in the first model (paratibial model). In addition, this model mimics the bone lesions observed in human.

### 3.9. Lung Metastasis Mouse Model

To study the effect of OS-EPS derivatives on the metastatic ability of osteosarcoma, 1.5 × 10^5^ POS-1 cells were injected by the technique of retro-orbital injection of the venous sinus [26]. Mice were anesthetized by inhalation of a combination isoflurane/air (1.5%, 1 L/min) and they received buprenorphine after the tumor cell injection (0.05 mg/kg; Temgesic^®^, Schering-Plough). A preventive protocol was established with four-week-old male C3H/HeN mice (*n* = 7 per group) divided into 3 groups: PBS, heparin and OS-EPS 15 kDa. A first subcutaneous injection (12 mg/kg of OS-EPS derivative or heparin in 50 µL of PBS) was performed 30 minutes before the POS-1 cell injection. Then, 4 subcutaneous injections were done daily at 6 mg/kg for the derivative or heparin. Mice were euthanized when mice showed signs of lung metastases development (respiratory distress, weakness, weight loss, and dorsal kyphosis). Lungs were collected for histological and macroscopic analysis: the lungs were categorized according to the size (big or small) of the metastases.

### 3.10. Statistical Analysis

All *in vitro* experiments were realized 3 times. Numbers of cells per field mean counts were compared by a non-parametrical Wilcoxon test. Mean tumor volumes were compared using Kruskal-Wallis test. The size of lung metastases categorical variable was analyzed by Fisher’s exact test. The difference was considered significant at *p* < 0.05.

## 4. Conclusions

Osteosarcoma is a rare malignant tumor of bone with dramatic clinical outcome. Indeed, lung metastases are frequent in the history of the diseases and are associated with a very high level of mortality. Polysaccharides are known to modulate numerous cell functions especially cell adhesion and migration and may be potentially interesting therapeutic approaches for treating patients suffering from cancers. In the present manuscript, we demonstrated the therapeutic interest of three oversulfated low molecular weight marine bacterial exopolysaccharides (OS-EPS). With their low efficiency in clotting assays and their ability to reduce the *in vitro* invasiveness of osteosarcoma cells as well as the metastatic process, OS-EPS represent new class of polysaccharides with high interest in oncology. In our study, only the OS-EPS 15 kDa derivative could effectively inhibit both migration and invasiveness of osteosarcoma cells *in vitro*. Moreover, the OS-EPS 15 kDa derivative was very efficient at inhibiting the establishment of lung metastases *in vivo*. Such polysaccharides could also be useful for developing new delivery systems for conventional chemotherapeutic agents, even if their mechanism of action is not yet known [37].

## Figures and Tables

**Table 1:** Molecular weight and chemical composition of the oversulfated exopolysaccharide (OS-EPS) derivatives.

EPS Derivatives	Mw * g/mol	Mn * g/mol	I * Mw/Mn	S ** %	Neutral Sugars *** %	Acidic Sugars **** %
OS EPS GYS15	16000	14000	1.14	15	18	10
OS EPS GYS8	10000	8000	1.25	13	19	12
OS EPS GYS4	5300	4700	1.13	14	23	11

* Mw = weight average molecular mass, Mn = number average molecular mass, I = polydispersity index (Mw/Mn); ** Sulfur was determined by elemental analyzer and HPAEC chromatography; *** Method of Dubois *et al.* (1956) and **** Method of Filisetti-Cozzi and Carpita (1991).

## Supplementary Materials

Supplementary materials can be accessed at: http://www.mdpi.com/1420-3049/21/3/309/s1.
