# Supplementary material for: Anti-Metastatic Properties of a Marine Bacterial Exopolysaccharide-Based Derivative Designed to Mimic Glycosaminoglycans
Source: Molecules. 2016 Mar 4;21(3):309. doi: 10.3390/molecules21030309 (PMC6273430; doi:10.3390/molecules21030309)
Supplement: Supplementary file 1 [file molecules-21-00309-s001.pdf]

Supplementary Materials: Anti-Metastatic Properties of a Marine Bacterial Exopolysaccharide-Based Derivative Designed to Mimic Glycosaminoglycans

Dominique Heymann, Carmen Ruiz-Velasco, Julie Chesneau, Jacqueline Ratiskol, Corinne Sinquin and Sylvia Colliec-Jouault

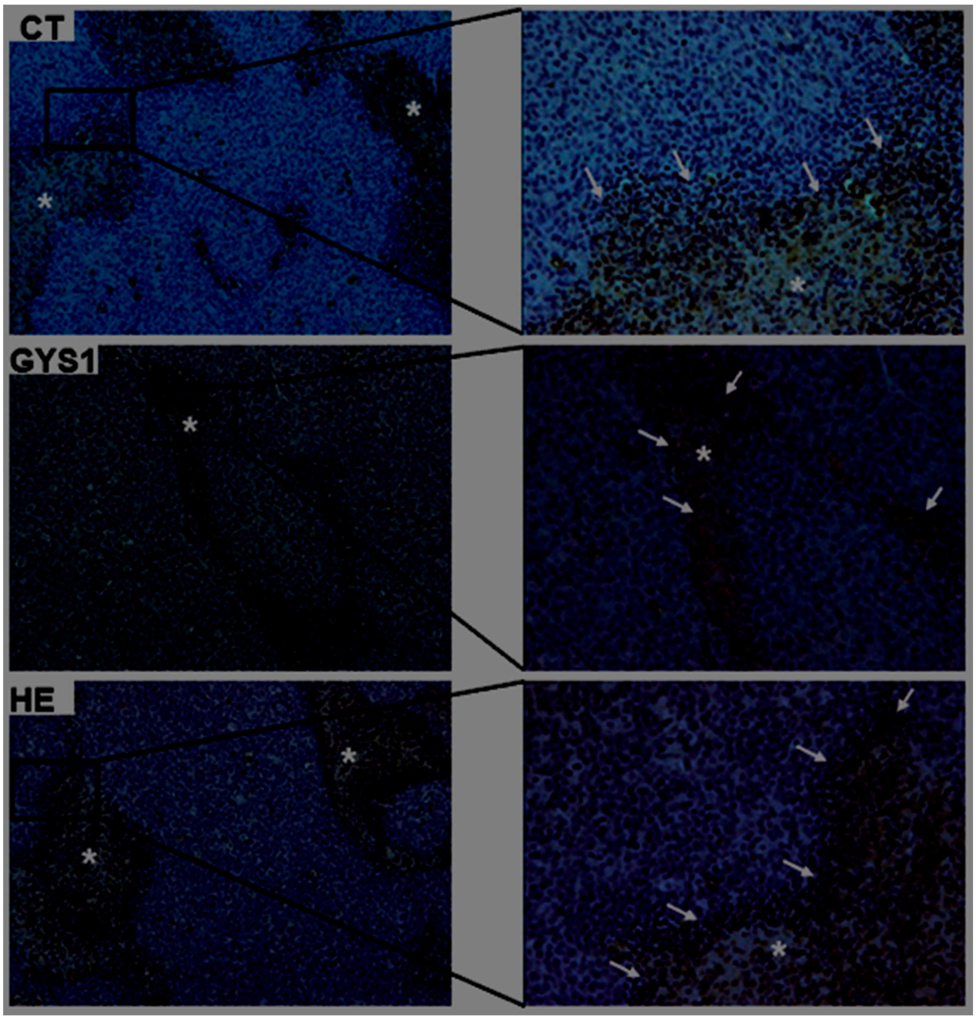

Figure S1. Oversulfated exopolysaccharide (OS-EPS) derivative did not induce pro-apoptotic effect compared to heparin and control group. Apoptotic cells in primary bone tumor were determined using the *in situ* cell death detection kit (Roche Diagnostics), based on the terminal-deoxynucleotidyl transferase–mediated dUTP nick-end labeling (TUNEL) method. Tumor masses were characterized by peripheral proliferation area centered by necrotic foci (*). Apoptotic cancer cells were observed at the border between proliferation and necrotic areas (arrow). Original magnification: ×100 (**right panels**); ×200 (**left panels**).
